# Editorial: Role of eye movements in vision, attention, decision-making, and disease

**DOI:** 10.3389/fpsyg.2024.1445167

**Published:** 2024-06-25

**Authors:** Kaveri A. Thakoor, Fatema Ghasia, Hantao Liu

**Affiliations:** ^1^Columbia University, New York, NY, United States; ^2^Cleveland Clinic, Cleveland, OH, United States; ^3^Cardiff University, Cardiff, United Kingdom

**Keywords:** eye movements (EM), artificial intelligence, Alzheimer's disease, medical expert decision-making, ophthalmology, non-human primates, eye tracking calibration, visual displays

Eye movements play a role in tasks as mundane as browsing an advertisement on the internet to as high-stakes as piloting an aircraft or performing a complex surgery. Technologies to probe and harness human eye movements for understanding internal state, deciphering decision-making mechanisms of experts, or elucidating mechanisms behind behavior abound in neuroscience, engineering, and medicine. This Research Topic compiles five articles that provide insights on the role of eye movements in medical-expert decision-making, reviews state-of-the-art technology for calibrating eye tracking technology, sheds light on underlying mechanisms behind behavior in response to rewarding stimuli, and even showcases how eye movements may be a window to cognitive state.

Hu et al. provide a study of the role of reward in smooth pursuit eye movements in non-human primates called, “*Sensorimotor-linked reward modulates smooth pursuit eye movements in monkeys*.” Smooth pursuits, like fixations and saccades, are a critical eye movement behavior that are employed by humans and non-human primates alike. However, their role in reward has not been fully examined. Authors of this article probe how smooth pursuits differ for non-human primates when they are given a juice reward vs. not given a juice reward. They find that there exist two unique mechanisms for the initiation of smooth pursuit vs. the steady-state continuation phase of smooth pursuit based on their study; this provides a deeper understanding of the role of reward and how it impacts these two distinct segments of smooth pursuit. This may have implications for human eye movements in reward situations as well.

Yamada et al. investigate differences in eye movements between two often-confused diseases of cognitive decline: Alzheimer's Disease (AD) and Lewy Body disease (LBD). By comparing features of eye movements in cohorts of subjects from these two populations, they identified disease-specific alterations in patterns of eye movements between the two groups. In AD individuals, they observed diminished visual exploration compared to matched controls, a characteristic associated with cognitive impairment, which is also found to correlate with motor impairment in LBD. They also observed reduced allocation of gaze to objects, which has been found to imply weaker attention to high-level/abstract image features in AD patients and enhanced image-center bias in LBD patients. These findings have the potential to assist in distinguishing AD from LBD and may enable care-givers to better understand how these two populations perceive the world and thus how to help provide meaningful interventions to enhance their quality of life especially for everyday activities involving vision.

Liu et al. contribute a comprehensive review of personalized gaze tracking calibration technologies that exist today entitled, “*A Review on Personal Calibration Issues for Video-Oculographic-Based Gaze Tracking*.” The authors quantitatively compare 2D and 3D gaze tracking approaches through simulation experiments. They highlight how different personal calibration settings impact performance. In this process, they introduce multiple important design issues that must be kept in mind when developing new personal calibration methods (beyond the standard 9-point calibration used in many devices today). This paper is one of the first of its kind to thoroughly review personal calibration modalities in existence with the goal of enhancing the ease of human-computer interaction during eye-gaze tracking in order to enable its more widespread adoption in engineering/medicine/neuroscience research.

Akerman et al. examine the eye movements of expert ophthalmologists vs. those of ophthalmologists-in-training as they view optical coherence tomography reports for the diagnosis of glaucoma. The authors find that experts tend to exhibit significantly fewer fixations and spend significantly less time on glaucomatous or healthy OCT reports compared to their novice counterparts to come to a diagnosis decision. They also find specific image/report-regions that are fixated more often by experts for the accurate diagnosis of glaucoma. Furthermore, the authors develop simple neural-network models to classify with high accuracy an expert image-viewer vs. a novice image-viewer, which could have significant implications for medical education (how to evaluate skill progression/acquisition) and on how ultimately blindness-causing eye diseases are diagnosed.

Kim and Yoshida explore how displays with varying resolution at center vs. periphery impact the sense of agency of viewers especially in the context of determining authorship. This research has major implications for how information provided to viewers can impact their decision-making and choices even in everyday settings like viewing a website, which in turn can impact sales and artistic choices and thus highly-lucrative advertisement industries.

Together the five articles herein provide a unique lens through which to view the state-of-the-art role of eye movements in vision, attention, decision-making, and disease. We anticipate the reader will take away knowledge on eye tracking technologies, the power of eye movements in multiple industries involving visual attention, and the scope for future exciting research employing eye movements as a window to external behaviors, medical-expert decision-making, and internal disease state.

## Author contributions

KT: Writing – review & editing, Writing – original draft. FG: Writing – review & editing. HL: Writing – review & editing.

